# CART Peptides and Drugs of Abuse: A Review of Recent Progress

**DOI:** 10.4303/jdar/235984

**Published:** 2016-06-28

**Authors:** Michael J. Kuhar

**Affiliations:** Yerkes National Primate Research Center, Emory University, 954 Gatewood Rd NE, Atlanta, GA 30329, USA

**Keywords:** drug abuse, addiction, CART peptide, cocaine and amphetamine regulated transcript, psychostimulants, alcohol, nicotine, opiates, nucleus accumbens, reward

## Abstract

Earlier studies suggesting an involvement of cocaine and amphetamine regulated transcript peptide (CARTp) in the actions of drugs of abuse are confirmed in the most recent publications. This seems especially true for the psychostimulants where CARTp in the nucleus accumbens inhibits or regulates the actions of these drugs; the regulation is lost after repeated drug use which may be an important mechanism in addiction. The other drugs, including nicotine, alcohol, opiates, and perhaps caffeine can affect CARTp or CART mRNA levels. While the exact mechanism is not always clear, the hope is that these findings may provide some insight for the development of medications. While binding studies indicate the existence of specific G-protein coupled receptors (GPCR) receptors for CARTp, major work to be done is the cloning of these receptors.

## 1. Introduction

This is a review of progress on the role of CART peptides specifically in the area of drug abuse. It covers the last five to six years, and the papers discussed were identified in a PubMed search using the terms “CART peptides (CARTp)” and “cocaine and amphetamine regulated transcript (CART).” In this review, the discussion is arranged according to the various drugs and CART receptors. For information on the discovery of CART, its genes and processing and regulation, receptors and signaling, and its role in a variety of functions and diseases, see other more general reviews and papers [1,2,3,4,5].

In the literature search, it was found that CART was implicated in a number of other topics that are not related to drug abuse and are not addressed here. These include feeding and body weight, endocrine regulation, stress, recovery after stroke, anxiety and depression, hypertension, general and comparative anatomy, and yet others. CART continues to be a very fertile area of research with many implications.

CART peptide will be abbreviated CARTp and it will refer to CART (55–102) or CART (62–102), or the corresponding peptides found in the human brain (CART (42–89) and (49–89)) [1].

## 2. Psychostimulants

The prominent hypothesis regarding CARTp and cocaine is that the peptide reduces the action of cocaine or dopamine, particularly in the nucleus accumbens (NAc), and that this is an important regulatory mechanism for the physiological effects of cocaine and dopamine. If cocaine produces an increase in locomotor activity (LMA), then the acute injection of CARTp into the NAc reduces the cocaine-induced LMA [1] as well as cocaine self-administration [6]. There do not seem to be sex differences in these effects [7]. Many recent studies reviewed below support this hypothesis.

Because injecting exogenous CARTp into the NAc reduces dopamine-related activity, it was reasonable to test the effect of reducing endogenous CARTp. This was done using short hairpin RNAs (shRNAs) against CARTp and scrambled shRNAs as controls. After bilateral injections of the shRNAs into the NAc, the animals exhibited an increase in body weight, and an increase in cocaine-induced LMA. These effects are opposite to those found with injection of the peptide into the NAc. Immunohistochemistry revealed a decrease in CARTp levels in the shRNA injected rats compared to controls [8]. Taken together, these findings support the hypothesis about CARTp’s regulation of cocaine’s effects.

Most of the experiments showing that CARTp reduced cocaine’s LMA effects were performed acutely, that is with acute cocaine and acute CARTp injection. An intriguing question was whether or not there were changes in these effects in rats given cocaine chronically (addicted?). In rats given cocaine for many days, the inhibitory effect of CARTp was lost suggesting that the process of dependence or addiction was accompanied by, or perhaps required, a loss in the regulatory role of CARTp on cocaine in the NAc [9]. It may not be surprising that the process of addiction is accompanied by a loss of negative regulatory effects within reward pathways.

A study by Moffett et al. [10] addressed the mechanism by which CARTp inhibited the actions of dopamine in the NAc. Specifically, the study asked which dopamine receptors produced the inhibitory effect. Injection of a D1 receptor agonist into the NAc led to an increase in LMA which was increased, not decreased, when CARTp was coadministered with the D1 agonist. There was no effect on LMA following coadministration of CARTp and D2 or D3 agonists. But simultaneous injection of D1 and D2 agonists into the NAc led to LMA greater than that obtained via administration of individual D1 or D2 agonists, and the LMA due to this combination was attenuated by intra-NAc CARTp. The inhibitory effect of CARTp was found only when both receptors were activated simultaneously.

Several experiments studied cocaine-related activity, but in other behavioral paradigms. Chronic exposure to cocaine results in enhanced dopamine signaling and increased CART expression in the NAc. These effects are thought to be related to the expression of behavioral sensitization. CARTp injection into the NAc results in several signaling changes in the cells. Peng et al. [11] showed that repeated injections of CARTp into the NAc reduced the cocaine-enhanced phosphorylation of dopamine receptors and signaling changes in the NAc. Also, the cocaine-induced behavioral sensitization was inhibited by repeated injection of CARTp into the NAc. Thus, it appears that CARTp inhibits changes in the dopamine receptors and their signaling which has behavioral consequences.

Along these lines, D1 (especially) and D2 antagonists blocked both cocaine-induced behavioral sensitization and also CARTp overexpression. D1 KO (knock out) mice also showed reduced cocaine-induced behavioral sensitization and CART overexpression. These results suggest that the D1 receptor plays an important role in regulating CART expression [12]. Yoon et al. [13] studied conditioned locomotion in rats treated with cocaine. CARTp injected bilaterally into the NAc inhibited the conditioned hyperlocomotion.

While it is known that cocaine and amphetamine can induce CART mRNA and CARTp levels, Cadet et al. [14] also found that methamphetamine (second injection) increased CART mRNAs in the NAc.

Several studies have elucidated the neuroanatomical location of the effects of CARTp. Almost all of the studies on CARTp over the last several years have focused on the NAc. But a few studies have examined other structures. The NAc projects to several structures including the ventral pallidum (VP). Using anterograde and retrograde pathway tracing, it was found that CART-containing neurons in the NAc project to the VP [15]. The CARTp-containing nerve terminals in the VP resemble GABAergic synapses, and injection of CARTp into the VP inhibited cocaine-induced locomotion. Thus CARTp might have a regulatory role in the action of cocaine in areas other than the NAc. In a similar study, projections from the paraventricular nucleus of the thalamus (PVT) were known to go to the NAc. Injections of CARTp into the PVT inhibited drug primed reinstatement of cocaine lever pressing. Injections around but not in the PVT were without effect [16]. The basolateral amygdala (BLA), which also receives projections from the PVT, has been implicated in psychostimulant-induced reward and aversion, and the results were dose-dependent. Intra BLA infusions of 2 *μ*g of CARTp produced conditioned place preference (CPP) while 4 *μ*g produced aversion (CPA). Low (subrewarding) doses of CARTp and low doses of amphetamine combined to produce CPP [17]. Thus, CARTp actions in the BLA can affect reward and amphetamine-induced reward. It appears that CARTp can work at several anatomical levels.

Most studies of CARTp involve direct injections of peptide into parts of the brain. But, there is also a study where CARTp was administered intraperitoneally (IP). Job and Kuhar [18] showed that CARTp injected IP inhibited both cocaine and amphetamine-induced LMA just as it does when given in the brain. The effect was biphasic having a maximal reducing effect at about 25 *μ*g/kg. This dose would elevate CARTp levels in the blood significantly, and some earlier work suggests that CARTp could cross the blood brain barrier and elevate brain CARTp levels [19]. Another study examined the effects of injecting purified single chain variable fragments (IP) from antibodies against CARTp [20]. Chai et al. [20] showed that IP administration of the antibody fragments reduced the increase in locomotor activity caused by repeated cocaine administration but had no effect on acute cocaine’s effects. The antibodies should reduce peripheral CARTp which is opposite to the effects of injecting CARTp as done by Job and Kuhar [18]. Without knowing what happens in the brain, the results of these studies can be difficult to interpret. Nevertheless, it seems that peripheral CARTp levels can produce some behavioral effects. Also, the idea that peripheral CART-related agents can have an effect on brain-behavior may have implications for treatments.

Volkoff [21] showed that CARTp had similar effects in goldfish as in mammals, with implications for the role of CARTp during the evolution of species. Hsieh et al. [22] provided evidence that oxidative stress in the brain is involved in CARTp’s regulation of appetite control in amphetamine-treated rats. Finally, CART gene expression in the NAcs of subgroups of Sprague-Dawley animals was not very predictive of addiction vulnerability, whereas properties of the CART system in Lewis rats might contribute to the vulnerability of this strain to drug-seeking behavior [23].

In summary, there have been many new studies of the interactions of CARTp and psychostimulants. Reducing CARTp levels in the NAc produces effects opposite to those found after injecting CARTp into the NAc. Importantly, after repeated cocaine treatments, and the animals may be dependent, CARTp has no effect. The loss of the regulatory action of CARTp after repeated drug administration may be part of an addicting mechanism. The effects of CARTp can be directly related to a simultaneous action at multiple dopamine receptors. CARTp also shows regulation when other more complex behavioral paradigms are used. In most studies, the NAc was a direct target. But other anatomical studies show that CARTp can have an effect in other anatomical regions that are connected to the NAc. Injections of CART into the periphery might also have central effects. Overall, these studies support the hypothesis that CARTp regulates psychostimulant and dopamine-related activities associated with the NAc circuitry.

## 3. Nicotine

Nicotine is one of the most abused substances in the world, and understanding the mechanisms involved has a high priority. In the time period under consideration for this review, several papers have appeared that show a connection between nicotine and CARTp. Dandekar et al. [24] examined the effect of CARTp on nicotine-induced anorexia and weight loss, and there were a number of findings. In an acute study, CARTp increased the anorectic effect of nicotine; in a chronic study, and after the cessation of nicotine administration, CARTp prevented the hyperphagia and weight gain found during nicotine withdrawal; acute nicotine produced an increase in CART-immunoreactive cells and fibers in the paraventricular nucleus (PVN) and arcuate; thus hypothalamic CARTp seems to be involved in the feeding and body weight effects of nicotine.

Kaya et al. [25] examined levels of CART mRNA and CARTp in various regions of the reward-related mesocorticolimbic system. After five days of administration of nicotine, various changes were found in various regions. There was a downregulation of CARTp in many of the regions and an interesting, functionally opposite upregulation of CART mRNA in some regions as well. It seems clear that nicotine can affect CARTp levels in reward-related areas.

In an interesting maternal study, Younes-Rapozo et al. [26] found that fourteen-day administration of nicotine to lactating rats resulted in a reduction in CART positive cells in the PVN in adulthood. Thus maternal nicotine can change hypothalamic peptides in adulthood and therefore have lasting effects. In a human study [27], there was no significant association in CART gene variants in schizophrenic patients who used alcohol or nicotine; thus CART may not play a role in schizophrenia comorbidity with nicotine or alcohol use.

In summary, CARTp has been associated with nicotine’s effects in feeding and body weight and in the regulation of reward-related brain regions. An important observation was that exposure of lactating rats to nicotine produced changes in CARTp in the brain when the offspring were adults. Such long lasting changes over the life span are amazing and significant from the perspective of factors that control vulnerability to drug use.

## 4. Alcohol

Alcohol, along with nicotine, is one of the most abused substances in the world. Given the important role of CARTp in regulating the mesolimbic system, it is reasonable to explore the role of CART in alcohol use and dependence. Alcohol administration increased CART mRNA levels in the NAc [28]. Using CART knockout (KO) mice, Salinas et al. [29] found that the KOs consumed less alcohol than the wild types (WT). Also, KO females showed a greater sensitivity to alcohol than the WTs.

Hypothalamic neurons that contain CARTp are activated by alcohol-related stimuli [30]. CARTp in the amygdala (central nucleus) may be involved in alcohol withdrawal mechanisms [31]. King et al. [32] focused on reinstatement of alcohol intake. They showed a role for CARTp in preventing reinstatement, suggesting a potential therapeutic role for CARTp and similar substances. Similarly, in a context-induced reinstatement paradigm, injections of CARTp into the NAc shell also attenuated drug-associated environment reinstatement [33]. In an opposite approach, quieting/inhibiting the NAc shell by injections of GABA agonists, reinstated extinguished alcohol seeking. Evidence was presented that the NAc shell mediates extinction by inhibiting hypothalamic peptidergic neurons [34]; this in turn promoted a hypothesis that neural circuits involved in satiety are involved in extinction training. These studies by McNally and colleagues were well controlled and focused on the important aspect of drug abuse in humans which is extinction and reinstatement. Chronic human drug abusers suffer repeated relapses which are modeled in animal studies as reinstatement.

In summary, these several recent studies show an involvement of CARTp in alcohol intake. Also, CART is involved in reinstatement of drug taking; reinstatement is an important model of relapse in humans.

## 5. Opiates

Opiates are prototypic, centuries old drugs of abuse. A possible involvement with CARTp is a therefore a natural question.

Salas et al. [23] showed that CART mRNA in the NAc was downregulated by acute morphine in rats. Bakhtazad et al. [35] examined levels of CART mRNA and peptide in male rats after administering morphine under several conditions: acute low dose, acute high dose, and chronic escalating doses. Also withdrawal was induced by injections of naloxone. CARTp and mRNA were increased in the high dose and in the withdrawal groups in the NAc, striatum, and prefrontal cortex (PFC). In the chronic cocaine group they were decreased in the NAc and striatum, and some additional changes were found. These results are clearly compatible with a view that CARTp is involved in the effects of morphine and that they may mediate some of these effects in reward-related anatomical areas. Further, as is mentioned above in Section 2, the effects of opiates on CARTp weaken when the drugs are used repeatedly [35].

Upadhya et al. [36] approached the question by considering a reward-related opioid-dopamine-mesolimbic pathway between the ventral tegmental area (VTA) and the NAc. Self-administration (SA) of food pellets, intracerebroventricular (ICV) injections and intra-NAc injections were utilized. ICV injection of CARTp increased food pellet SA. Also, intra-NAc shell injection of CARTp increased food pellet SA, while CARTp antibody caused the opposite. The effects were specific for the NAc because intra-PVN injections had no effect. Injection of morphine into the NAc shell increased SA and pretreatment with CARTp increased SA further. Intra-NAc injection of CARTp antibody reduced the reinforcing effects of morphine. Administration of a DA agonist (IP) increased SA but intra-NAc injection of a CARTp antibody blocked the effect of the DA agonist. There are CARTp neurons in the NAc, and thus CARTp seems to act downstream of DA.

The role of opioids on gene expression was examined by Anghel et al. [37]. Morphine was administered either for short term or long term and many different transcripts were up- or downregulated. CART mRNA was one of them. Baldo et al. [38] examined peptides related to reward modulation by sleep loss. Rats were sleep deprived for 10 days and CART mRNA was downregulated in the arcuate nucleus. These studies show that CARTp is involved in the effects of opiates in food-related areas which are also involved in reward.

As shown for other drugs, opiates interact with and produce changes in CART in reward-related brain regions as well as in food-related areas. An interesting finding was that the effects of opiates on CART were reduced with chronic opiate use; this is similar to what was found with stimulants. It is not surprising that with chronic drug use or addiction, there would be a loss of regulatory mechanisms that normally were in place to control the effects of the drugs.

## 6. Caffeine

Caffeine (CAFF) is a substance known to affect addicting substances and behavior while not being considered addicting in itself. Recently, Job [39] found that while intra-NAc CARTp did reduce cocaine-induced LMA as expected, it had no effect on CAFF-induced LMA. Because it had been proposed that CAFF-induced LMA did not involve increased levels of DA in the synapse, Job concluded that CARTp blunts the effects of drugs when DA is involved and not otherwise.

Hu et al. [40] tested whether repeated CAFF administration would cause behavioral sensitization and an increased expression of CART. It did produce behavioral sensitization, and CART levels did increase and peaked at day 5 and then declined. The changes in CART expression were altered by a variety of receptor blockers including those for adenosine receptors as one might expect. Thus, CAFF, perhaps the most used psychoactive substance in the world, also affects CARTp systems. Again, the response was biphasic suggesting that CART’s involvement changes depending on the duration of use or dose of the drug. It would have been interesting to test if intra-NAc injections of CARTp had an effect on the behavior caused by repeated CAFF injections.

## 7. CARTp receptors

The receptors for CARTp are a final, needed part of the CART story. While there are no reports of cloned genes for the peptide, binding studies show the existence of a CARTp receptor. Lin et al. [41] studied PC12 cells and carried out binding with radio labeled CART (61–102). Specific binding increased with differentiation of the PC12 cells and this was inhibited by actinomycin D and cycloheximide. CARTp treatment of the cell membranes increased the binding of [S-35]GTP gamma S indicating that the binding site for CARTp was a g-protein related receptor. Also, CARTp elicited increased phospho-ERK and it was blocked by pertussis toxin suggesting a link to Gi/o proteins. Also, PACAP (6–38) was suggested to be a CARTp receptor antagonist.

Nagelová et al. [42] also studied PC12 cells and found that CART binding increased after nerve growth factor-induced differentiation. Dexamethasone treatment which transforms PC12 cells into chromaffin-like cells reduced CARTp binding. CART peptide itself did not seem to cause differentiation by its lack of effect on acetylcholinesterase. CARTp treatment increased phosphorylation of SAPK/JNK and subsequent c-jun protein expression; inhibition of JNK-kinase reversed these effects. Central administration of CARTp increased c-jun immunoreactive cells in areas related to food intake, the dorsomedial nucleus of the hypothalamus (DMH), and nucleus of the solitary tract.

Another study bears on the CARTp receptor. Blechová et al. [43] carried out a structure-activity study of CARTp. CARTp is known to have three disulfide bridges, and they systematically altered these bridges. They found that the disulfide linkage between cysteines 68 and 86 in CART (61–102) was not important for biological activity but the other two bridges were required. Thus a specific structure is required for receptor activation.

Interestingly, there is evidence that CARTp directly binds to subunit B of the mitochondrial enzyme known as succinate dehydrogenase [44]. Because this target is subcellular, it is also possible that CARTp may act at intracellular receptors/targets.

These studies do not show complete agreement in all details, but overall they show that a CARTp receptor is a GPCR with Gi/o coupling. Also, the conformation of CARTp is restricted at the receptor. Cloning of the receptor(s) is an important goal.

## 8. Conclusions

Evidence for connections between many drugs of abuse and CARTp is abundant. But the degree of impact or influence of endogenous (vs. exogenous) CARTp on behavior is not always clear. There are some uncertainties such as whether or not the injections of exogenous CARTp produce the same effects as natural CARTp neurotransmission. There may also be some technical problems. For example, are the effects of injections misleading because of diffusion to distant sites? Many of these key findings need to be replicated. Almost all the studies rely on measuring the levels of the peptide or mRNA and it is not always clear that changes in peptide or mRNA are significant in producing behavior. Also, with regard to measuring levels, turnover studies [45] could be more informative but these are not yet undertaken. In spite of the problems and uncertainties, the overall, repeated, supportive findings are convincing. CART is found in reward-related brain regions and drugs of abuse activate the CART system. The major work to be done is to identify and clone the receptors for CARTp. This would result in a great advance in CARTp biology.
